# Editorial: Testicular Cancer: New Insights on the Origin, Genetics, Treatment, Fertility, General Health, Quality of Life and Sexual Function

**DOI:** 10.3389/fendo.2020.00041

**Published:** 2020-02-07

**Authors:** Andrea Garolla, Ugo De Giorgi, Domenico Milardi

**Affiliations:** ^1^Unit of Andrology and Reproductive Medicine, Department of Medicine, University of Padova, Padova, Italy; ^2^Department of Medical Oncology, Istituto Scientifico Romagnolo per lo Studio e la Cura dei Tumori (IRST) IRCCS, Meldola, Italy; ^3^Unit of Endocrinology, University Policlinic Gemelli, Rome, Italy

**Keywords:** testis cancer, genetics, male infertility, sexual function, cancer treatment

About 95% of all testicular cancers are represented by testicular germ cell tumors (TGCTs), which include seminoma and non-seminoma histological types. TGCT is the most common solid tumor among males 15–34 years of age, with an estimated 8,850 new cases and 410 deaths during 2017 in the United States. The highest incidence rates of testicular cancer are in Norway (11.8 per 100,000) and the lowest are in India (0.5 per 100,000) and Thailand (0.4 per 100,000). The annual incidence of Testicular cancer (TC) has doubled over the past 40 years with an increasing trend over time, particularly in Caucasian males (1).

The pathogenesis of TC is poorly known. The identification of pathogenic mechanisms and risk factors involved in testicular carcinogenesis still represent topics of extremely high clinical interest. The origin of TGCT, probably starting at early stages of embryogenesis, seems to be a part of the Testicular Dysgenesis Syndrome (TDS) where some early PGC/gonocytes are blocked in their differentiation, are tightly regulated by epigenetic modification in terms of microRNA expression and DNA methylation, retaining their early marker profile (Baroni et al.).

It is now clear that genetic, environmental and hormonal risk factors concur and mutually influence both the development of the disease and its prognosis. Indeed, the probability of developing TC is the result of a combination of a number of factors that can be distinguished into genetic, environmental and hormonal factors. The most common risk factor for testicular cancer is undescended testis (cryptorchidism); others risk factors are personal or family history of testicular cancer, age, ethnicity, and infertility.

Moreover, a possible causal relationship between viral infections and TGCTs was firstly evoked almost 40 years ago and is still a subject of debate. Recent efforts in oncological and virological research have brought to light the oncogenic potential of different virus species. Evidences from a systematic review and meta-analysis support an oncogenic effect of HIV and EBV on the human testis, but the evidence was insufficient to establish causality (Garolla et al.). Therefore, the exposition to different environmental agents, such as pesticides and non-steroidal estrogens can increase the risk of developing this neoplasm. The increased exposure to environmental factors, particularly chemical pollutants with endocrine disrupting activity, can alters the major hormonal axis that drives testis development and function from gestational age. The susceptibility to these alterations further depends on genetic factors that justify the strong familiarity of TC (De Toni et al.). The presence of testicular microlithiasis in patients with such risk factors increases more the risk of cancer. Testicular microlithiasis (TM) represents itself a risk factor for TGCT, because in infertile men the presence of TM is associated to an ~18-fold higher prevalence of testicular cancer as reported in a full meta-analysis of eight carefully selected studies (Barbonetti et al.). Longitudinal studies are warranted to elucidate whether this cross-sectional association actually reflects a higher susceptibility of infertile men with TM to develop testicular cancer over time.

This special issue, entitled “Testicular Cancer: New Insights on the Origin, Genetics, Treatment, Fertility, General Health, Quality of Life and Sexual Function,” provides an overview of clinical diagnosis and disease management and an approach to explain the molecular development of TC. The limited number of studies and the resulting lack of exact knowledge about development, differentiation, and treatment of TC leaves several clinical problems regarding treatment and follow-up unsolved. The aim of this special issue intends to give an update on the most controversial issues in research areas of TGCT, as well as new results and express the opinions of a selection of specialists who have expanded the field with their recent discoveries. Both clinical and basic researches are reported and many questions are addressed. One of these deals regard the diagnostic strategies which still remain problem to be solved. Diagnosis for TGCTs is greatly based on detecting serum markers such as alpha- fetoprotein, beta-human chorionic gonadotropin, and lactate dehydrogenase but only 60% of all patients show elevations of these markers. For this reason tumor markers alone are not able to detect many recurrences, indeed in about 40% of men with disease recurrence the levels of these markers are usually “normal.” Therefore, the discovery of novel clinical biomarkers of TC would clearly help the early detection and the monitor of the disease. The use of proteomic platforms permit to discover putative prognostic and diagnostic markers of testicular cancer. A panel of proteins for early detection, identified by proteomics technique, might be used for prognostic evaluation and for follow-up of TC. Moreover, the molecular mechanisms revealed by these proteomic studies might represents molecular targets for anticancer treatments (Milardi et al.).

With effective treatment, the overall five-year survival rate of TGCT is 97%. Men diagnosed with GCT have excellent survival rates due to advances in the multimodal treatment paradigm of chemotherapy, radiation therapy, and surgery. Despite the good response of these tumors to platinum-based chemotherapy, some patients are refractory to treatment and present poor clinical outcomes. During carcinogenesis and tumor development, cancer cells reprogram energy metabolism toward a hyper-glycolytic phenotype, with over-expression of metabolism related proteins, like glucose and monocarboxylate transporters, pH regulators, and intracellular glycolytic enzymes (Warburg effect). The alterations in the expression of proteins related with the Warburg effect and hyper-glycolytic and acid-resistant phenotype are associated with aggressive clinicopathological parameters (Bonatelli et al.). Other molecular findings are also associated with local cancer invasiveness as OCT4, KLF4, and PTTG1 expression in seminoma (Grande et al.).

Retroperitoneal Lymph Node Dissection (RPLND) is generally considered as a treatment option for non-seminomas, when lymph nodes are compromised. There are three different RPLND techniques: open, laparoscopic, and robotic. The open approach is as effective as the other two in its oncological efficiency. Recent studies have been pointing out a slight increase of advantages on the robotic approach. Also, it is noteworthy that new technologies are on the rise, improving the laparoscopic approach, requiring further studies after their uses are consolidated (Vaz et al.).

Testicular function usually gets worse after treatment for testicular cancer, so the patients must be carefully followed for signs of hypogonadism and associated comorbidities. Hypogonadism has been often reported in testicular tumor survivors because of the radio- and/or chemo-induced Leydig cell damage. Longitudinal studies have revealed a higher negative impact of chemotherapy on Leydig cell function than radiotherapy or orchiectomy alone, leading to a higher risk for hypogonadism and its related complications, including cardiovascular, metabolic and bone mineralization impairment, and sexual dysfunction in testicular tumor survivors. Compared to orchiectomy alone, combined or high-dose chemotherapy and radiotherapy increase the risk for metabolic syndrome, DM, and cardiovascular events (La Vignera et al.).

These cancer survivors will therefore have to live with the long-term physical and psychological consequences of both their treatments (surgery, chemotherapy, radiotherapy) and the diagnosis itself. Invasive and destructive surgery such as retroperitoneal lymph node dissection increases the frequency of such dysfunctions (2). However, reports of thorough longitudinal follow-up from diagnosis to long-term survivorship are rare and confirm that TC and its treatment have a significant effect on sexuality. The absence of a clear correlation with biochemical hypogonadism suggests that this may to a large extent be due to the surgical procedure itself, or to the psychological impact of a cancer diagnosis (Pallotti et al.).

Individual health, sexual relationships affect several important aspects of survival and significantly influence the QoL of long-term survivors. Physical, psychological, work-related problems and changing perspectives about work and life in general influenced life and career decisions among testicular cancer survivors (Schepisi et al.).

Finally cancer treatment is not an individual experience, but induces deep effects on patients' families, who often have to assume a caregiving role for the duration of and following treatment for cancer. The role of a more integrated system of the patient and his social support, with the purpose of improving QoL not only during active treatment, but also in the follow-up period, and to encourage a less traumatic return to the everyday life (De Padova et al.).

We would like to express our sincere gratitude to all authors and referees for their contribution to this issue. Multidisciplinary and collaborative efforts in recent years have clearly improved our understanding of the pathogenesis of testicular cancer but this disease remains an enigma in many aspects especially the environmental etiology.

## Author Contributions

All authors listed have made a substantial, direct and intellectual contribution to the work, and approved it for publication.

### Conflict of Interest

The authors declare that the research was conducted in the absence of any commercial or financial relationships that could be construed as a potential conflict of interest.
