# Corrigendum: Editorial: Testicular Cancer: New Insights on the Origin, Genetics, Treatment, Fertility, General Health, Quality of Life and Sexual Function

**DOI:** 10.3389/fendo.2021.712499

**Published:** 2021-06-10

**Authors:** Andrea Garolla, Ugo De Giorgi, Domenico Milardi

**Affiliations:** ^1^ Unit of Andrology and Reproductive Medicine, Department of Medicine, University of Padova, Padova, Italy; ^2^ Department of Medical Oncology, Istituto Scientifico Romagnolo per lo Studio e la Cura dei Tumori (IRST) IRCCS, Meldola, Italy; ^3^ Unit of Endocrinology, University Policlinic Gemelli, Rome, Italy

**Keywords:** testis cancer, genetics, male infertility, sexual function, cancer treatment

## Incorrect Affiliation

In the published article, there was an error in affiliation 2. Instead of “Department of Medical Oncology, Scientific Institute Romagnolo for the Study and Treatment of Cancer (IRST) IRCCS, Meldola, Italy”, it should be “Department of Medical Oncology, Istituto Scientifico Romagnolo per lo Studio e la Cura dei Tumori (IRST) IRCCS, Meldola, Italy”.

The authors apologize for this error and state that this does not change the scientific conclusions of the article in any way. The original article has been updated.

